# Building quality culture through accreditation: a PRECEDE-guided exploration of leadership in clinical units

**DOI:** 10.1108/IJHCQA-10-2025-0161

**Published:** 2026-01-08

**Authors:** Maria Pilar Mesa‐Blanco, Pedro E. Ventura-Puertos, Víctor M. González-Chordá, Manuel Rich-Ruiz

**Affiliations:** Maimonides Biomedical Research Institute of Cordoba (IMIBIC), Córdoba, Spain; University Hospital Reina Sofía, Córdoba, Spain; University of Córdoba, Córdoba, Spain; Interdisciplinary Research Group in Discourse Analysis (HUM380), Córdoba, Spain; Department of Nursing, Pharmacology and Physiotherapy, University of Córdoba, Córdoba, Spain; Nursing Research Group (GIENF-281), Universitat Jaume I, Castellón de la Plana, Spain; Investén-isciii, Madrid, Spain; Biomedical Research Networking Centre in Respiratory Diseases (CIBERES), Madrid, Spain

**Keywords:** Hospitals, Leaders, Organisational culture, Qualitative research, Quality, Transformational leadership

## Abstract

**Purpose:**

This study aims to explore the perceptions of clinical management unit leaders regarding their role in healthcare accreditation processes within a European public hospital. It aims to identify the predisposing, enabling and reinforcing factors that shape leadership practices and influence the development of a quality-oriented organisational culture.

**Design/methodology/approach:**

A qualitative hermeneutic approach was employed, combining individual and group semi-structured interviews and participant observation. Six healthcare professionals responsible for accreditation projects under the Andalusian Agency for Health Quality participated. Data were analysed using Ricoeur’s interpretative framework, guided by the PRECEDE model.

**Findings:**

Three themes emerged in this study: (1) conceptual tensions between accreditation and quality culture, (2) leadership as a collective and emotionally demanding endeavour and (3) accreditation as a process that generates both tangible improvements and emotional reinforcement. The study highlights the need to clarify the internal lead’s role and align accreditation language with clinical and managerial realities.

**Research limitations/implications:**

Future research could examine how gender dynamics influence leadership in accreditation processes.

**Practical implications:**

Findings support the need for leadership development, institutional investment and the redesign of accreditation standards to better reflect clinical practice and managerial responsibilities.

**Social implications:**

The study underscores the emotional and cultural impact of organisational change, highlighting the importance of supportive environments that foster shared responsibility, team engagement and sustainable quality improvement.

**Originality/value:**

This is the first study to apply the PRECEDE model to explore leadership in accreditation. It offers a novel framework for understanding how internal leads navigate quality improvement within complex healthcare organisations and provides actionable insights for institutional development.

## Introduction

According to Donabedian (2005), evaluation is a fundamental basis for measuring the quality of care. To assess the quality of services provided, it is essential to understand the dimensions and values of quality care for stakeholders, as well as the reference standards that guide professionals towards quality practice (Donabedian, 2005; Marcomini *et al.*, 2022). In response to the need to define and evaluate care quality, The Joint Commission (2022), an accreditation agency in the United States, was established. Since then, such agencies have proliferated, and various accreditation models have been developed (The Joint Commission, 2022), including that of the Andalusian Health Quality Agency (ACSA) (Agencia de Calidad Sanitaria de Andalucía, 2022), a public organisation serving one of Spain’s most populous regions.

For ACSA, accreditation is a strategic resource that enables healthcare units to pursue continuous improvement, fostering a dynamic cycle of self-assessment aimed at excellence, with active engagement from all stakeholders (Agencia de Calidad Sanitaria de Andalucía, 2017). Its model provides a conceptual framework to guide those responsible for Clinical Management Units (CMUs), encouraging reflection on the unit’s starting point and offering reference standards and tools to support the development of improvement plans (Agencia de Calidad Sanitaria de Andalucía, 2019). Currently, around 400 CMUs are accredited by ACSA (Fundación Progreso y Salud *et al.*, 2022).

Each accreditation project has an internal person appointed by the unit director. Regardless of their role within the CMU, this individual is responsible for coordinating the unit’s self-assessment for external accreditation. However, this role is complex, as it is not clearly defined within the ACSA model. More importantly, there is limited evidence regarding the experiences of these project leads, including their coping strategies, needs, expectations, and challenges throughout the accreditation process. This gap underscores the relevance of our study, which draws on the dimensions of the PRECEDE model (Cilliers *et al.*, 2015; Green and Kreuter, 1991), outlined in Box 1.

Box 1 **Description of the PRECEDE model**

The PRECEDE model is a behavioural diagnostic framework used to identify the factors that influence whether a behaviour is adopted or maintained. It is commonly applied in the health field to promote adherence to healthy behaviours and to discourage risky ones. The model categorises influencing factors into three groups:

1. Predisposing factors: These relate to motivation and are more subjective in nature. They include knowledge, beliefs, values, attitudes, and perceived needs that influence an individual’s or group’s inclination to act.
2. Enabling factors: These refer to the resources, skills, or conditions that facilitate the performance of a behaviour once motivation is present. They include access to tools, organisational support, and structural conditions.
3. Reinforcing factors: These appear after the behaviour has been performed and take the form of rewards or punishments. They include emotional, social, or material feedback that strengthens or weakens the likelihood of repeating the behaviour.

A thorough analysis of the predisposing, enabling, and reinforcing factors associated with their leadership role will support institutional managers, unit governance, and support services in selecting the most suitable profiles, providing leadership training for improvement projects, enhancing support tools, and offering more proactive guidance. This approach will help optimise resource use and maximise outcomes in the pursuit of excellence in healthcare delivery.

The general objective of this study is therefore to understand the perceptions of leaders of CMU accreditation projects regarding their role in the accreditation process, identifying their predisposing factors (motivation, expectations, training/perceived needs, beliefs, and values), distinguishing their enabling factors (tools and tangible resources), and recognising their reinforcing factors (emotional, material, and social reinforcements, as well as satisfaction with the outcome).

## Method

This study employs a qualitative methodology grounded in a phenomenological–interpretative paradigm (Merriam and Tisdell, 2016). The rigour and quality criteria applied were those proposed by Calderón (2002) as demonstrated in Table 1.

**Table 1:** Rigour and quality criteria

Criteria	Description
Theoretical, epistemological, and methodological soundness	A hermeneutic approach is well suited to exploring perceptions, values, and expectations in context—key elements of quality culture. It enables rich interpretation of quality-related phenomena beyond traditional biomedical standards
Relevance	The predisposing, enabling, and reinforcing factors identified shape internal leads’ behaviour and support the embedding of quality culture. Understanding these dynamics enhances the transferability of findings to similar healthcare settings
Validity	Triple triangulation was applied: (1) between the lead author and participants, (2) between the lead author and the research team, and (3) across data collection methods (interviews and observation)
Reflexivity	The lead researcher has worked for three years in the Quality Unit of the institution where the study participants are based. Her role includes advising units undergoing self-assessment for accreditation, making her the team member with the closest working relationship to internal leads. Although she knows all participants personally, she has only worked directly with four. She is familiar with the content of each project, its accreditation level, the profile of each internal lead, and the maturity level of each unit in terms of quality improvement
	This proximity to the phenomenon under study provided strong motivation to undertake the research, with the aim of generating findings that could facilitate the accreditation process and ultimately enhance care quality within the institution
	The remaining team members are academics with expertise in both qualitative and quantitative research, and are also involved in quality accreditation processes within their respective universities

The study was conducted in a tertiary-level university hospital within the Andalusian Health Service, characterised by a high level of care complexity.

Participants were healthcare professionals internally responsible for ACSA accreditation projects within CMUs. A purposive sampling strategy was used, specifically an intensity approach (Merriam and Tisdell, 2016). The estimated sample size was six leaders, with the final number determined by discourse saturation—understood to occur when interviews no longer yielded new or relevant insights into the phenomenon (Baker *et al.*, 2012). Recruitment was carried out by the lead author, who contacted participants by telephone to arrange the date, time, and location of the interview, according to each participant’s convenience.

Data were collected in June 2023 by the lead researcher through five semi-structured interviews—four individual and one group interview. The latter was a triangular interview (Merriam and Tisdell, 2016) involving two participants. All interviews followed the same script, as outlined in Table 2.

**Table 2:** Semi-structured interview script

Opening questions
*Could you tell me which accreditation projects your unit is currently involved in?*
Questions primarily exploring predisposing factors
*What does leading an accreditation process mean to you, and what do you consider its key moments?*
*What motivated the initiation of the accreditation process?*
*What motivated you to take on the role of internal lead for the accreditation process?*
*What are your expectations regarding your unit’s accreditation?*
*Did you feel you had the necessary skills to lead this project?*
Questions primarily exploring enabling factors
*What has helped you in this process, and how?*
*What has hindered your leadership, and in what ways?*
*We’ve already discussed some difficulties—can you describe any others you encountered during the self-assessment?*
*What resources and tools have you had access to for the accreditation process?*
Questions primarily exploring reinforcing factors
*How satisfied are you with the outcome of the evaluation?*
Mixed or cross-cutting questions
*What needs did you identify during the self-assessment?*
*What has been your experience with the tools provided?*
*What helped you move forward during the accreditation process?*
*Does the evaluation outcome reflect your perception of your unit’s reality? Why or why not?*

Interviews lasted between 40 and 90 min, were conducted face-to-face, audio-recorded, and transcribed using Microsoft Word 2016.

Additionally, four naturalistic participant observation sessions (Merriam and Tisdell, 2016) were conducted during self-assessment meetings in two CMUs during the same period. These sessions involved two internal accreditation managers, unit leadership, other team members, and the lead author, who acted as an advisor.

As part of the validation process, interview transcripts were returned to participants, who were invited to review and clarify their contributions.

To ensure anonymity and confidentiality, participants were identified using a letter system. Each was assigned the letter “L” followed by the chronological number of their interview. In group interviews, the letter “G” preceded the “L”. This system was also applied to quotes or content from participant observation, using the acronym “OP”.

In line with the study’s paradigm, Ricoeur’s framework for hermeneutic analysis was followed (Tan *et al.*, 2009). The dimensions of the PRECEDE model (Cilliers *et al.*, 2015; Green and Kreuter, 1991) served as initial analytical categories. NVivo 14 software (Paulus *et al.*, 2017) was used throughout all stages of the analysis.

In the preliminary phase, the lead author conducted a thorough reading of the first transcribed interview (L1), identifying its overall meaning and compiling an initial list of ideas to form an intuitive explanatory framework. The analysis of verbal and non-verbal narratives then proceeded through three levels (Tan *et al.*, 2009). In the first (Explanation), complete meaning units (or nodes) related to the initial categories were selected. Sixteen analytical subcategories were created, each linked to one of the starting categories. In the second (Naïve comprehension), nodes were reorganised into these subcategories and then grouped into codes that conveyed more abstract meanings while qualifying different aspects of the same idea. At this stage, the researcher began coding the remaining interviews, linking new nodes to existing codes or creating new ones as needed. In the third stage (In-depth understanding), a deeper interpretation of the leaders’ perceptions was achieved through the “hermeneutic arc”, allowing for the highest level of abstraction. This process led to the development of patterns encompassing main categories and subcategories. A second team member conducted a parallel analysis to triangulate the findings. At each stage, decisions were discussed with the research team and participants. The final explanatory framework was agreed upon and used to guide the discussion of findings.

The research team adhered to the principles of the Declaration of Helsinki (The World Medical Association, 2021), and the study received approval from the provincial research ethics committee (code number 5440).

## Results

The sample consisted of six participants. The first four subjects participated in individual interviews, while GL5 and GL6 participated in the group interview. The sociodemographic characteristics are presented in Table 3.

**Table 3:** Sociodemographic description of the interviewees

ID	Gender	Age	Professional category	Occupation	Years as an internal manager	Years of professional experience
L1	M	68	Physician	CMU Director	12	35
L2	F	48	Physician	Specialist Physician Quality and Patient Safety Supervisor	3	23
L3	M	47	Nurse	Nurse in Charge	4	26
L4	M	35	Physician	Specialist Physician	3	10
GL5	F	56	Physician	CMU Director	6	31
GL6	F	59	Physician	CMU Director	6	34

**Figure 1:**
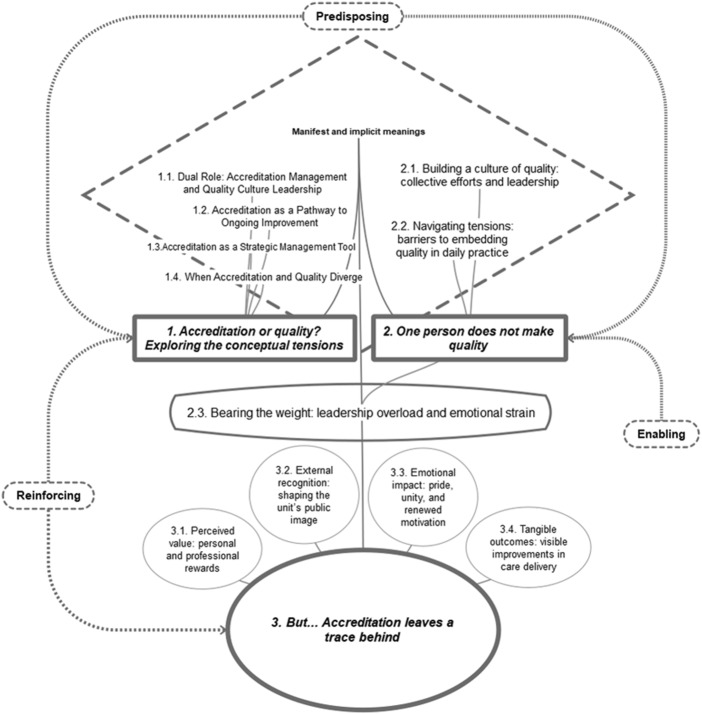
Explanatory framework of results with patterns and categories

The analysis identified the following themes, depicted in Figure 1.

The participants’ predisposing, enabling, and reinforcing factors are collected in Table 4.

**Table 4:** Predisposing, enabling, and reinforcing factors

Factors	Role of the internal lead in the accreditation of a clinical management unit	
	In favour	In opposition
Predisposing	Accreditation perceived as a tool for continuous improvement and strategic management Quality culture ◦ Continuous improvement identified as the primary motivation ◦ Presence of a committed/engaged team ◦ Quality culture perceived to spread organically at both the micro (CMU) and macro (institutional) levels	Absence of a shared project Negative perceptions and attitudes within the team Cultural dissonances ◦ Use of inaccessible language ◦ Quality not perceived as part of the professional identity ◦ Accreditation perceived as additional or unnecessary work Emotional strain and stress
Enabling	Quality culture ◦ Established body of knowledge through training and experience ◦ Availability of tools and resources (certifying agency, General Services, Quality Unit) ◦ Presence of a trained and engaged team In-house training initiatives Use of management and leadership tools Institutional support and commitment	Actions perceived as superficial or symbolic (“for showing”) Difficulty understanding and applying manual requirements and standards Limitations related to ICT. Bureaucratic burden Care overload limiting available time and energy Lack of time and space for sharing experiences and information Accreditation-related tasks often completed outside regular working hours
Reinforcing	Satisfaction with the accreditation outcome Material reinforcement (e.g. resources, staffing, or recognition) Social reinforcement (e.g. team recognition, institutional visibility) Emotional reinforcement (e.g. pride, motivation, sense of achievement) Perceived advancement in the unit’s quality culture	Dissatisfaction with the accreditation outcome Material reinforcement expected but not received

The themes mentioned in Figure 1, along with their corresponding categories and subcategories, will be described in narrative form below. Illustrative participant quotes supporting each thematic category are provided in Supplementary File 1.

### Accreditation or quality? Exploring the conceptual tensions

In addition to serving as internal managers of the accreditation project, participants were directly involved in care quality, assuming varying degrees of responsibility within their units. As a result, during the interviews, accreditation and quality management were often intertwined—and at times indistinguishable. When prompted, participants described this dual role as a combination of project management and promoting a quality-oriented culture.

All participants understood accreditation as a tool for continuous improvement. When asked about her motivations for initiating the process, L2 responded: “Then you see that part of your work […] you see some benefit, don’t you? Well, we are delighted with …”—followed by a list of improvements achieved in her unit. Continuous improvement and care quality were frequently cited as the main drivers behind the accreditation process. Several participants even described ongoing improvement as their primary goal, emphasising its direct impact on patient care.

Participants also recognised accreditation as a management tool—a framework for assessing the unit’s current state, defining future goals, and outlining a roadmap to achieve them.

However, participants were clear that accreditation does not always equate to quality—and vice versa. They identified areas that had been addressed solely to meet accreditation requirements, yet failed to translate into meaningful changes in practice. Additionally, varying levels of satisfaction were expressed regarding the accreditation achieved and its alignment with their perceived reality, suggesting that the accreditation–quality relationship was not always seen as coherent.

### One person does not make quality

In this theme, the development of a quality culture was described as an ongoing project that placed a significant burden on team leaders. Informants acknowledged the need to foster and sustain this culture within their units. Although they did not offer a formal definition, they identified several key components: a body of knowledge to be acquired; a distinct language that required effort to master; a set of effective support tools—including intra-hospital collaboration, General Services, and specific digital applications; and, crucially, people. Human resources were seen as essential, particularly those with a deeper understanding of quality, informatics, and office systems, and a high level of engagement.

To foster this emerging culture, internal managers developed training programmes to support the adoption of the unit’s working methods. Building this collective project required involving a broad range of staff as active participants in the accreditation process and as promoters of the quality culture. Both self-assessment and external evaluation were seen as essential for driving continuous improvement. In this way, the culture gradually took root—*seeping into every corner of the organisation*, as GL5 described—until the unit itself began to demand further growth in this way of working.

However, participants also described the quality culture as a work in progress, and identified *cultural dissonances* between this ideal and the day-to-day reality of their units. The first major culture shock stemmed from difficulties in understanding the requirements of the accreditation process. These challenges were linked to three core issues:

1. *I don’t understand it, so I need someone to translate it for me.*
2. *I understand the standard, but I don’t know how to demonstrate it.*
3. *Some standards feel disconnected from the clinical reality of my unit.*

Participants also noted that the quality culture was not perceived as a shared project within their units, suggesting that it was not part of their organisational DNA. According to informants, this lack of “anchoring” in quality practices highlights the need for targeted training—particularly at early stages of professional development, such as undergraduate education and residency.

However, the challenges extended further. Participants noted that quality-related tasks were often perceived as external to the core responsibilities of healthcare professionals—seen as an added burden rather than an integral part of clinical work. This perception was reinforced by the already substantial demands of direct patient care.

Among the negative attitudes towards accreditation, the most extreme position was held by team members who neither understood its purpose nor participated in care improvement efforts, and in some cases, actively hindered the accreditation process.

Participants also identified additional barriers to developing this collective project, including staff shortages, job insecurity, limited time and space for collaboration, and care overload. As a result, those most directly involved in the accreditation process often had to complete related tasks outside of their regular working hours.

A common request among participants was for stronger institutional support. All interviewees emphasised the importance of unit leadership allocating both human and material resources to facilitate the accreditation process. In some instances, middle managers attempted to recognise staff engagement by incorporating their contributions into performance evaluations, thereby framing participation in a more positive and motivating light. This alignment between quality improvement efforts and broader management objectives led L1 to question whether responsibility for both should rest directly with unit leadership.

Another recurring issue was the presence of shortcomings related to ICTs. Field diary entries frequently highlighted a lack of digital competencies among staff, as well as the significant amount of time required for training in the use of basic office tools—such as word processors, spreadsheets, document management systems, and clinical information systems—as well as the platform used by the certifying agency.

The dual responsibility of managing both accreditation and fostering a quality culture contributed to a strong sense of overload among unit leaders. All reported feeling overwhelmed by the demands of their roles. They acknowledged the significant bureaucratic workload involved, which consumed considerable time, resources, and energy. In this regard, GL5 and GL6 noted that strictly adhering to the accreditation process would require dedicating their entire working day to it. In some cases, this responsibility had been delegated—or even imposed—on them, which further intensified negative perceptions of the process’s more bureaucratic aspects.

As a group, participants bore the brunt of the stress and emotional exhaustion generated by the demands of the accreditation process and their limited control over external factors. They frequently described their work using language that conveyed sustained tension and relentless effort, and acknowledged experiencing emotional highs and lows—often expressing frustration at their inability to meet all objectives. In response to this overwhelming pressure, they turned to management tools to identify areas requiring support, prioritise tasks, and delegate responsibilities more effectively.

### But … Accreditation leaves a trace behind.

Participants questioned the ultimate value of accreditation processes, both for themselves and for their units. While they acknowledged that accreditation could bring material benefits—such as intensified research activity (e.g. more protected time and resources) and improved coordination with certain support services—they expressed disappointment that other forms of recognition or reward were, at least initially, absent from their own or their team’s expectations.

They acknowledged the role of accreditation in shaping the unit’s public image. However, they did not consider this the most meaningful benefit, nor did they view it as the primary reward of the evaluation process.

On the other hand, participants described achieving accreditation as a source of pride—for both the team and themselves. This pride in their collective accomplishments fostered a strong sense of unity within the unit.

However, what stood out most was that participants observed tangible improvements in the unit’s care—small changes that gradually took place and became consolidated over time. They identified several of these now-established improvements, including enhanced organisation, reduced variability through the implementation of procedures and protocols, improved management of adverse events, and more accurate completion of clinical records.

## Discussion

This study explores the perceptions of healthcare professionals responsible for leading an accreditation process within a Clinical Management Unit, under the framework of the Health Quality Agency of Andalusia (southern Spain). Three main themes emerged from their accounts. First, the tension between accreditation and quality stood out, highlighting accreditation as a tool for continuous improvement and strategic management, while also revealing that it does not always equate to genuine quality. Second, participants described building a culture of quality as a collective endeavour that requires the engagement and support of multiple actors. When this support is lacking, the burden falls disproportionately on internal managers, leading to work overload and emotional strain. Finally, despite these challenges, participants recognised meaningful incentives in their work—particularly the tangible outcomes of improved care and small, lasting changes—which provided emotional satisfaction and a sense of purpose.

Regarding the first theme (*Accreditation or quality? Exploring the conceptual tensions*), the ACSA defines the internal project manager as a person designated by the institution seeking accreditation, whose role is to facilitate the development of the process and ensure communication between the unit and the Agency. However, the specific responsibilities associated with facilitating the process are not clearly defined. This lack of clarity contributes to a blurring of roles in participants’ accounts, where the functions of the project manager often overlap with those of quality management.

The assumption of leadership in the accreditation process was evident across the cases studied. However, the development of project management competencies—considered essential by leading institutions such as the Institute for Healthcare Improvement (2025) in the United States—was often overlooked. These competencies, along with so-called soft skills, which Goldman and Wong (2020) identify as core to quality and safety improvement, play a crucial role in driving meaningful change in healthcare settings.

Two key factors were identified as facilitators of the internal manager’s engagement in the accreditation process. First, the instrumental nature of accreditation—as a tool for strategic management and continuous improvement—is well documented in the literature (Carrasco-Peralta *et al.*, 2019; Ellis *et al.*, 2020). Second, continuous improvement itself was perceived as an expected and desirable outcome of the process (van Bodegom-Vos *et al.*, 2017). This predisposition was further reinforced when the manager and their team had a prior track record in quality initiatives and had already witnessed tangible results, acting as a positive motivator for continued involvement.

However, as our findings illustrate, accreditation is not always synonymous with quality—and vice versa. While both participants and the literature acknowledge the positive outcomes associated with accreditation processes (Alkhenizan and Shaw, 2011; Basarah *et al.*, 2022; Dutra and Guirardello, 2021; Underwood *et al.*, 2020), they also point to discrepancies between accreditation status and actual quality performance, as highlighted by Swathi *et al.* (2020) and the Accreditation Association for Ambulatory Health Care (2024).

This tension between formal accreditation and perceived quality has prompted international agencies to rethink their models. In 2025, The Joint Commission (2025) has introduced *Accreditation 360*, a redesigned framework that eliminates over 700 outdated requirements and reorganises standards into 14 National Performance Goals. The model claims to offer continuous engagement, making standards publicly accessible, to reduce bureaucratic burden and better align accreditation with clinical realities.

Concerning the second theme (*One person does not make quality*), our findings highlight the centrality of quality culture in participants’ discourse. From an anthropological perspective, the concept of acculturation offers valuable insights into how cultural change can be facilitated (Choi *et al.*, 2019). Raghunathan (2012) in his study on the implementation of checklists in clinical settings, reflects on the role of cultural dissonance in communication breakdowns. He argues that these challenges were overcome through the assimilation of professionals into the new culture. This form of assimilation—characterised by a diminished emphasis on preserving one’s original cultural framework and an active effort to adapt to the new one (Choi *et al.*, 2019), —enabled more fluid and direct communication (Raghunathan, 2012).

The level of acculturation within participants’ CGU varied considerably. Leaders were tasked with bridging a gap between a more traditional, conservative, and continuity-oriented culture and a more dynamic, critical culture focused on continuous improvement. Facilitating this cultural shift requires *intercultural mediation* (De and Souza, 2016) supported by the soft skills previously mentioned (Antonacci *et al.*, 2018), which are essential for effectively disseminating and embedding quality improvement practices.

The first cultural dissonance that accreditation process leaders must address is the belief that quality is not embedded in the professional or organisational DNA. As several authors have noted, this perception is closely linked to the lack of formal training in quality and safety during undergraduate and postgraduate education (Alhawajreh *et al.*, 2023; Bendermacher *et al.*, 2020, 2021; Bethune *et al.*, 2013; Davey *et al.*, 2022).

Another cultural dissonance identified was the perception of quality-related tasks as “extra work” that competes with time allocated to direct patient care. This perception reinforces resistance to quality initiatives. However, quality management methodologies such as Lean have been shown to streamline processes and potentially free up time for bedside care—or for other activities professionals deem necessary (Hamilton *et al.*, 2014; nhs.uk, 2022; Wilson, 2009).

New technologies also play a critical role in the transition toward a quality-oriented culture. However, when these tools fail to align with the actual needs of the organisation, they can become barriers rather than facilitators. This concern, echoed in our findings, is also supported in the literature (White *et al.*, 2016).

One of the most significant challenges reported was the lack of resources, time, and capacity to implement improvement plans. This often forced internal managers to carry out bureaucratic tasks outside of regular working hours—a situation also noted by White *et al.* (2016) and Vaughn *et al.* (2019).

According to participants, the effort required to engage the team in the project—combined with the burden of bureaucratic tasks resulting from limited team involvement—led to stress and emotional exhaustion (Alshamsi *et al.*, 2020). While the literature includes studies on leadership-related stress and its negative impact on team dynamics (Cheung *et al.*, 2019; Harms *et al.*, 2017; Klebe *et al.*, 2022; Parent-Lamarche and Biron, 2022), the emotional experiences of those leading quality improvement efforts remain underexplored. This gap underscores the relevance of our study as a novel contribution to the field.

In the final theme (But … Accreditation leaves a trace behind), the discussion centres on the concept of reinforcers—factors that contribute to the perceived or actual reward experienced by those involved in the accreditation process (Cilliers *et al.*, 2015; Green and Kreuter, 1991). Participants’ accounts revealed three types of reinforcers: emotional, social, and material.

Emotional reinforcement was reflected in expressions of joy, hope, and satisfaction upon receiving accreditation. As Álvarez-la Trinchera (2014), notes, “A leader is a specialist in the management of emotions—both their own and those of others.” In this sense, we argue that emotional management should be considered a core leadership competency. Harnessing this wave of positive emotions can help improve the emotional climate within the unit and act as a predisposing factor for reinforcing the shift toward a quality-oriented culture.

Social reinforcement was linked to the external recognition that accreditation confers (Alhawajreh *et al.*, 2023; Carrasco-Peralta *et al.*, 2019). Once accreditation is granted, organisations are authorised to use the Agency’s official brand (Agencia de Calidad Sanitaria de Andalucía, 2019), which serves as a symbol of distinction and credibility in the eyes of external stakeholders.

Material reinforcement included tangible benefits such as intensified research activity (e.g. more protected time and resources) and improved coordination with other services. Most importantly, accreditation was seen as instrumental in identifying and implementing improvements in care quality—an outcome also emphasised by Carrasco-Peralta *et al.* (2019).

Among the various dimensions of care quality, patient safety emerged as the most developed within the units studied. This finding aligns with existing literature (Carrasco-Peralta *et al.*, 2019; Kwan *et al.*, 2021), as does the reported improvement in clinical outcomes (Dutra and Guirardello, 2021), organisational order, reduction in practice variability, and more accurate record-keeping (Carrasco-Peralta *et al.*, 2019).

Our findings contribute to understanding a phenomenon that warrants greater clinical and academic attention. The emergence of quality culture as a central element offers an opportunity to deepen the synergies with disciplines such as anthropology, sociology, and transcultural nursing. The study also underscores the need to clarify the functions and competency map of the person responsible for the accreditation project. Furthermore, it aligns with the proposal to approach quality research from paradigms beyond quantitative designs (Singer and Vogus, 2013). Lastly, the innovative use of the PRECEDE model as an investigative tool linked to accreditation and quality improvement represents a unique and valuable contribution to the field.

The main limitation of the study is the absence of a gender perspective, which leaves unexplored how this variable could act as a predisposing, enabling, or reinforcing factor in accreditation leadership.

## Conclusions

This study highlights the complex and often under-recognised role of internal leads in healthcare accreditation processes. Far from being a purely administrative function, this role involves navigating organisational dynamics, fostering a culture of quality, and managing emotional and operational demands. Accreditation is perceived not only as a strategic tool for continuous improvement but also as a marker of organisational maturity.

The findings reveal that leadership in accreditation is shaped by uneven assimilation of quality principles, varying levels of team engagement, and limited institutional support. These conditions contribute to work overload and emotional strain, underscoring the need for clearer role definitions, targeted leadership development, and more integrated support structures within healthcare organisations.

Despite these challenges, internal leads identify meaningful reinforcements—emotional, social, and material—that validate their efforts and contribute to sustained improvements in care quality and patient safety. These outcomes suggest that accreditation, when aligned with clinical realities and supported by inclusive leadership practices, can serve as a catalyst for organisational learning, cultural transformation, and quality assurance.

By applying the PRECEDE model, this study offers a transferable framework for understanding the behavioural and contextual factors that influence leadership in quality improvement. It invites healthcare organisations to rethink how they select, support, and empower those leading accreditation efforts, recognising their strategic value in shaping a sustainable quality culture.

## Supplementary Material

Data supplement 1

## Notes

The supplementary material for this article can be found online.
