# Supplementary material for: Building quality culture through accreditation: a PRECEDE-guided exploration of leadership in clinical units
Source: Int J Health Care Qual Assur. 2026 Jan 8:1–15. doi: 10.1108/IJHCQA-10-2025-0161 (PMC12775687; doi:10.1108/IJHCQA-10-2025-0161)
Supplement: Data supplement 1 [file ijhcqa-10-2025-0161_suppl1.docx]

**Supplementary File 1. Illustrative Quotes by Thematic Category**

This file contains a selection of anonymised participant quotes, organised by the main themes and subcategories identified in the analysis. The quotes provide additional context and support for the findings presented in the Findings section.

**1. Accreditation or quality? Exploring the conceptual tensions**

- 1. *Dual Role: Accreditation Management and Quality Culture Leadership*

“If accreditation processes didn’t exist, everything would be left to the discretion of whoever is in charge of the unit. [...] With accreditation, you’re subject to quality control and to meeting certain standards.” L3

“One thing I’ve learnt is that when I see something wrong, I write it down, I report it, I ask questions. [...] It’s made me more aware that there really is a way for things to improve.” L4

“Getting others to do their part—not just the clinical work but the quality-related tasks—is very difficult.” L2

*1.2. Accreditation as a Pathway to Ongoing Improvement*

"To keep improving—but not for any other reason. It’s simply about being better." GL5

"… I don’t do it for the accreditation; I do it because I believe it’s in the best interest of the patients." L2

*1.3.Accreditation as a Strategic Management Tool*

“It helped me become more aware that there really is a way for things to improve—and that if things are already going well, they can stay that way.” L4

*1.4. When Accreditation and Quality Diverge*

"I think there are things that are going to be more for playing to the gallery, and I think there have been a lot of things for the gallery that those are the things that will cost a lot to improve because the dynamic in that sense, I think, is not good." L4

"Yes, in a high percentage, yes. [...] Basically, yes, they are reflected in a high percentage. The degree of satisfaction is high, isn't it?" L3

"It doesn't represent what we do here regarding quality, not at all. We have received a modest rating for the level of quality we provide in our service. To be honest." L2

**2. One person does not make quality**

*2.1. Building a culture of quality: collective efforts and leadership*

"Add to that the fact that you have to generate a culture of quality and work based on that. It's difficult, but it's done. But one person can't do it alone. You have to create that culture in everyone." L2

"I also received training. I did the *Expert Degree in Quality and Patient Safety in a Public Health Institution* in 2014. Anyway, from then on, more training, work, and gaining experience." L3

"The first time wasn’t easy at all… until you are assimilating and understanding that language, eh, because they have a language, well, they have their *own* language, quality has its *own* language." L3

"In general, it has been much easier, and that is because of the Quality Management Unit, which has given us a lot of facilities." GL6

"So, there is so much work, so much work, the coding of the equipment, the delegation of maintenance, of electromedicine, that of…, we work a lot with the General Services Department, a lot, so that helps you." L3

"What has helped us? Well, we have very involved personnel. Excellent staff, very involved" L2

"We have had a meeting to explain, we have had, uh, I have it here, [laughing] in which we have described, we have described the way the unit works, both for nurses and assistants, right?" L3

"Because, of course, if you want change, in the end you have to step out of that cycle everyone’s caught in and start bringing people along." L4

"Evaluation is *absolutely* essential… because if we don’t assess ourselves, both internally and externally, then unfortunately, we begin to decline — in terms of quality and in terms of patient safety." L1

"Those who were already doing things well kept doing them well, encouraging others to do the same, making sure everything was completed properly, labelled, and nothing left lying around. And those who weren’t doing things properly, well, in the end, they felt pressured — ‘everyone’s doing it this way, I guess I’ll have to do it like that too." L4

*2.2. Navigating tensions: barriers to embedding quality in daily practice*

"I don’t think it’s in most people’s DNA — no, it’s not. That is, self-criticism and self-evaluation still don’t seem to be sufficiently embedded in our daily practice" L1

"We didn’t learn about quality in a university classroom […] We learned it the hard way — by breaking our backs" GL5

"Because they see it as pointless work. The fact that you have to record things they see as unnecessary. Just extra work that keeps them from finishing their tasks on time" L2

"People see the computer as the enemy, because their priority is caring for patients. Writing things down is just as important as doing them — but the patient still needs to be looked after, you know what I mean?" L3

"And, obviously, just like anywhere else, they have had their black sheep too." L4

"What happens? Staff come and go, and that’s when we have to start all over again." L2

"It’s really hard for people to willingly give up time with their family or their free time… just to get involved in this." L4

"In fact, the only leverage I had was that every time I told someone, ‘Hey, look, we need to get this done…’, I’d CC the boss."" L4

"In the end, he’s their supervisor — and if he tells them they need to do something or keep an eye on something, they just do it." L4

"Yes, yes, I include it for them… there’s a goal, because within their individual objectives, I make sure they all actively contribute to the unit’s accreditation process every year." L3

"I believe that technology should make our work easier, but sometimes, it does just the opposite." L2

"That’s where you run into more difficulties — there’s a bit of a grey area when it comes to the IT side of things." L4

"And, like everything, some things work well and others don’t. But the support system — especially the maintenance side — is a weak spot, definitely a weak spot." L2

*2.3. Bearing the weight: leadership overload and emotional strain*

"When I first started, I was about to quit the hospital — I thought this just wasn’t for me..." L4

”But maybe what we need to do is write it down. And that means I’d have to be the one to write it. But honestly, I just don’t have the time — not humanly." L2

"Honestly, for me, it’s just been extra workload — a real burden." L4

"Yes, definitely — there have been areas where I’ve needed help. And obviously, right? I think that’s fundamental: recognising that you can’t do it all." L1

"So I set some priorities." L2

"What really made things easier for me was my colleagues' work and being able to delegate a large part of the responsibilities." L4

**3. But... Certification leaves a trace behind**

*3.1. Perceived value: personal and professional rewards*

"Yes, for example, there are many things—we could give specific examples. But generally speaking, starting an accreditation process in a unit opens doors and speeds everything up, for the better." L3

"And I think this should also be reflected in the healthcare system itself, particularly in terms of autonomy, as you progress through the levels of quality accreditation. They should, so to speak, grant you more freedom in decision-making. But in reality, this has no impact—at least none that is visible.." L1

"[…] physicians get intensifications, but in nursing there’s nothing—zero. So it’s really the way we work that keeps us going, that motivates us. But I don’t know… it would be a plus, right? Some kind of incentive—maybe financial, or something else, you know? Like inviting us to a conference, a quality-focused one or something, just to add a bit of spark, you know?" L3

*3.2. External recognition: shaping the unit’s public image*

“Therefore, this should indeed be a criterion for society when choosing—a criterion for feeling safe, for approaching care with greater peace of mind. I do believe it serves a purpose. But, as I said, it is more a consequence of what should already be part of our everyday practice.” L1

“Well, it’s the image you project of your service...” L2

*3.3. Emotional impact: pride, unity, and renewed motivation*

"Yes, that’s true, but I think it injects a sense of excitement." L1

"Especially because they’re genuinely happy about it, and it’s true—they do get involved." L1

*3.4. Tangible outcomes: visible improvements in care delivery*

"So things are gradually improving—little by little—in all areas of the unit, right?" L3

"That gives you—well, it gives you a sense of security and consistency in criteria […] You follow a procedure, and that helps avoid variability. It’s… And everything that’s written down and agreed upon leads to fewer errors. But that’s always been the case—fewer errors." L2

"I truly believe in the intrinsic value of the entire process and the journey itself. I mean, right now… yes, regardless of the score we’re given, I honestly think I’d do it all over again." GL5
